# Dietary protein intake during pregnancy and birth weight among Chinese pregnant women with low intake of protein

**DOI:** 10.1186/s12986-022-00678-0

**Published:** 2022-07-05

**Authors:** Jiaomei Yang, Qianqian Chang, Xueye Tian, Binyan Zhang, Lingxia Zeng, Hong Yan, Shaonong Dang, Yue-Hua Li

**Affiliations:** 1 Department of Epidemiology and Health Statistics, School of Public Health, Xi’an Jiaotong University Health Science Center, Xi’an, Shaanxi China; 2 Department of Obstetrics and Gynaecology, The First Affiliated Hospital of Xi’an Jiaotong University, Xi’an, Shaanxi China; 3 Nutrition and Food Safety Engineering Research Center of Shaanxi Province, Xi’an, Shaanxi China; 4 Key Laboratory of Environment and Genes Related to Diseases, Xi’an Jiaotong University, Ministry of Education, Xi’an, Shaanxi China; 5 Fourth Department of General Surgery, The Second Affiliated Hospital of Xi’an Jiaotong University, Xi’an, Shaanxi China

**Keywords:** Birth weight, Fetal growth, Maternal protein intake, Pregnancy

## Abstract

**Background:**

Previous studies have yielded inconsistent results on the association between maternal dietary protein intake and birth weight. Moreover, little is known about the effects of dietary protein intake from different sources on fetal growth. This study aimed to investigate the associations of different dietary protein sources (total protein, animal protein, plant protein, and major dietary protein sources) during pregnancy with birth weight and the related adverse birth outcomes.

**Methods:**

7310 women were recruited using a stratified multistage random sampling method at 0–12 months (median: 3; 10–90th percentile: 0–7) after delivery in Shaanxi, China. Maternal diets were gathered by a validated FFQ and other characteristics were collected by a standard questionnaire. Multilevel linear or logistic regression models were used to estimate birth weight changes or ORs (95% CIs) for adverse birth outcomes associated with different dietary protein sources during pregnancy.

**Results:**

The mean percentage of energy from total protein was 11.4% (SD 2.2), with only 27.4% of total protein derived from animal protein. Per 3% increase in energy from total protein, animal protein, and dairy protein was associated with birth weight increases of 19.4 g (95% CI 6.0–32.9), 20.6 g (4.8–36.5), and 18.2 g (4.7–31.7), respectively. Per 3% increase in energy from total protein, animal protein, and dairy protein was also associated with lower risks of low birth weight (LBW) (total protein: OR = 0.78, 95% CI 0.64–0.94; animal protein: 0.79, 0.65–0.96; dairy protein: 0.71, 0.56–0.91), small for gestational age (SGA) (total protein: 0.88, 0.79–0.98; animal protein: 0.87, 0.78–0.97; dairy protein: 0.81, 0.68–0.96), and intrauterine growth retardation (IUGR) (total protein: 0.84, 0.72–0.98; animal protein: 0.86, 0.75–0.98; dairy protein: 0.78, 0.66–0.92). We observed no associations of plant protein and other major dietary protein sources with birth weight and the above birth outcomes. The results did not change when maternal protein was substituted for fat or carbohydrate.

**Conclusions:**

Among Chinese pregnant women with low intake of protein, higher intake of dietary protein, in particular animal protein and dairy protein, is associated with higher birth weight and lower risks of LBW, SGA, and IUGR.

**Supplementary Information:**

The online version contains supplementary material available at 10.1186/s12986-022-00678-0.

## Background

Birth weight is an important indicator for fetal growth. Adverse birth weight, especially the related adverse birth outcomes, such as low birth weight (LBW), small for gestational age (SGA), and intrauterine growth retardation (IUGR), could influence not only perinatal mortality and morbidity but also the cardiometabolic health in adulthood [1]. Some studies have indicated that mother’s excess body weight in pregnancy can influence offspring weight through dietary habits and gut microbiota [2]. However, the underlying mechanisms of fetal growth have not been fully elucidated. Therefore, it is important to identify modifiable risk factors to provide evidence for the primary prevention of adverse birth weight.

Maternal nutrition during pregnancy, as an important modifiable factor, is critical for fetal growth. Among the nutrients studied, protein in particular appears to play a major role for fetal growth. Animal studies have shown that both insufficient and excessive dietary protein intake during pregnancy produced offspring with low birth weight [3]. However, findings from human observational studies on the effect of dietary protein intake during pregnancy on birth weight are inconsistent. Some studies showed a positive association between maternal protein intake and birth weight [4–7], while other studies reported no significant association [8–11], an inverse association [12–14], or even an inverse U-curve association [15]. To our knowledge, evidence on the independent effect of dietary protein intake during pregnancy on birth weight is scare in China, where maternal diets during pregnancy are typically monotonous and predominantly plant-based with little consumption of animal-based foods, especially in Northwest China [16, 17].

The primary sources of dietary protein are animal-based foods and plant-based foods [18]. Previous evidence have suggested that protein actions may vary by the amino acid types and food sources [19, 20]. However, the measurement of dietary protein intake during pregnancy has been mostly limited to total protein intake in the published human studies about birth weight and the related adverse birth outcomes. Only a few studies in Western countries have evaluated the associations of dietary protein intake from different sources during pregnancy with fetal growth [6, 21, 22]. To our knowledge, evidence on the effects of different dietary protein sources during pregnancy on birth weight and the related adverse birth outcomes is scare in Asian countries including China, where dietary habits, lifestyle factors, and fetal growth characteristics differ considerably from those in Western populations.

The present study aimed to explore the associations of different dietary protein sources (total protein, animal protein, plant protein, and major dietary protein sources) during pregnancy with birth weight and the related adverse birth outcomes (LBW, SGA, and IUGR) in Shaanxi Province of Northwest China.

## Methods

### Study design and participants

Details of the study design have been reported previously [17, 23]. In brief, a population-based cross-sectional study was performed in Shaanxi Province of Northwest China between August and November 2013. This area is normally divided into three regions: northern, southern, and central Shaanxi, with natural resources, culture, and lifestyle differing greatly among them. A total of 30,027 women who were pregnant during 2010–2013 were recruited using a stratified multistage random sampling method. The sampling process is as follows: twenty counties and ten districts were randomly sampled according to the proportion of rural to urban residents, population size, and fertility rate in Shaanxi, China; in each sampled county, six villages each from six townships were randomly selected; in each sampled district, six communities each from three streets were randomly selected; 30 and 60 eligible women were randomly selected from each selected village and community, respectively. Among the participants, 7750 women who were pregnant during 2012–2013 and had infants less than 12 months old were further interviewed to report their diets during pregnancy. We excluded 87 women with a multiple gestation, 65 women without offspring birth weight, and 288 women with an implausible total energy intake (less than 500 kcal/day or greater than 5000 kcal/day), leaving 7310 eligible participants for the final analysis. The flow diagram of sampling strategy with exclusion criteria is shown in Additional file 1: Fig. S1.

This study was conducted according to the guidelines laid down in the Declaration of Helsinki and all procedures were approved by the Xi’an Jiaotong University Health Science Center. Written informed consent was obtained from all participants.

### Maternal dietary assessment

Maternal dietary intake during the whole pregnancy was collected by a 107-item semi-quantitative food frequency questionnaire (FFQ) at 0–12 months (median: 3; 10–90th percentiles: 0–7) after delivery [17, 23]. Maternal dietary patterns and nutrient intakes tended to change little from early to late pregnancy [24]; thus, for large-scale epidemiological studies, especially for those with multiple dietary exposures and outcomes like the present study, diet assessment during the whole pregnancy at one time was reasonable, convenient, and economical [17, 23, 25]. The FFQ was established according to the previously validated FFQ designed for pregnant women in Shaanxi, China [26]. In the validation study, the Pearson correlation coefficient for protein between the FFQ and the average of six 24-h recalls was 0.61, with a range of 0.53 to 0.70 for other nutrients [26]. The frequency scales of five food items (animal oils, vegetable oils, salt, sugar, and sauce) were open-ended, and were listed as kilograms per month and the number of people regularly consuming them. The frequency of the other 102 food items was reported according to eight predefined categories ranging from never to two or more times per day, and their portion sizes were recorded according to food portion images [17, 23]. Daily intakes of total dietary protein, animal protein, plant protein, major dietary protein sources, and other nutrients were transformed using the China Food Composition Tables [27, 28]. Animal protein was derived from animal-based foods, including pork, beef, lamb, chicken and other poultry, eggs, dairy, fish, and seafoods. Plant protein was derived from plant-based foods, including cereals, legumes, nuts, vegetables, and fruits. The recommended percentages of energy from protein, fat, and carbohydrate in China were 10–20%, 20–30%, and 50–65%, respectively [29]. The recommended additional caloric intake per day during the first, second, and third trimesters of pregnancy in China was 0, 300 kcal, and 450 kcal, respectively [29].

### Birth outcomes assessment

Neonatal information including birth weight, gestational age, sex, and birth date was obtained by reviewing birth certificates. Birth certificates were finished by the medical staff once the neonates were born. Birth weight was measured with a baby scale with precision to the nearest 10 g. Gestational age at delivery was calculated according to the last menstrual period, and was confirmed by ultrasound scans. Medical records including physical examinations, clinical diagnosis, and medical history were referred to ascertain birth outcomes. The primary outcome of the present study was birth weight, and the secondary outcomes were LBW, SGA, and IUGR. LBW was defined as birth weight < 2500 g. SGA was defined as birth weight below 10th percentile of the gestational age-sex specific international reference for fetal growth [30]. IUGR was defined as birth weight below 3rd percentile of the gestational age-sex specific international reference for fetal growth [30].

### Covariates assessment

The general information of the participants during pregnancy was collected face to face by well-trained interviewers using a standard questionnaire. The study information was classified as follows: (1) socio-demographic characteristics: geographic area (northern, southern, or central Shaanxi); residence (rural or urban); childbearing age (< 25 years, 25–29 years, or ≥ 30 years); maternal education (primary school or below, junior high school, or senior high school or above); maternal occupation (farmer or working outside); nulliparity (yes or no); (2) health-related characteristics: passive smoking (yes or no); alcohol drinking (yes or no); antenatal care visit frequency (< 6 or ≥ 6); folate/iron supplements use (yes or no); anemia (yes or no); medication use (yes or no). Passive smoking was defined as being exposed to other people's tobacco smoke for ≥ 15 min/day. Alcohol drinking included a wide range of alcoholic beverages (liquor, wine, and beer) consumed in pregnancy. Folate/iron supplements use was defined as taking dietary supplements containing folate or iron for more than 2 weeks. Anemia in pregnancy was diagnosed using the criteria of hemoglobin concentration < 110 g/L. Medication use was defined as taking any medication in pregnancy.

### Statistical analyses

Because total energy intake is correlated with most nutrients, macronutrient intake was expressed as a percentage of total energy intake by the nutrient-density method and other nutrients were energy-adjusted by the residual method [31]. The study population characteristics according to quartiles of total dietary protein and animal protein intakes were described as percentages or means, with differences tested by χ^2^ test for categorical variables and analysis of variance for continuous variables. Household wealth index was established by principal component analysis according to the items reflecting family economic level (housing condition, vehicle type, income source, and type and number of household appliance), and this index was divided into thirds as an indicator for the poor, medium, and rich households [32]. To avoid multicollinearity of nutrients in regression analyses, we extracted the first component by principal component analysis according to the intakes of potential nutrients (vitamin A, thiamin, riboflavin, folate, vitamin C, vitamin E, calcium, zinc, and selenium) that explained 67.5% of the total variance, with the factor loadings of zinc, riboflavin, folate, thiamin, calcium, and selenium above 0.80.

Considering the stratified multistage random sampling design, multilevel models were applied to assess the associations of dietary protein intake with birth weight and the related adverse birth outcomes (LBW, SGA, and IUGR). After running the four-level empty models representing county (district)-township (community)-village(street)-individual, we observed nonsignificant within-group variations (all *P* > 0.05) and low intra-class correlations (all lower than 0.001) of the village (street) level; thus, simplified multilevel models with a random intercept at the county (district) and the township (community) levels were adopted. Multilevel linear regression models were used to estimate birth weight changes associated with different dietary protein sources during pregnancy, and multilevel logistic regression models were used to evaluate ORs (95% CIs) for adverse birth outcomes associated with different dietary protein sources during pregnancy. Protein intake values were computed as 3% energy units to assess the associations. The 3% energy unit was chosen because it was identical around 60 kcal energy and 15 g protein in the study population, which could be easily realized by increasing 75 g pork lean or 60 g chicken breast according to the China Food Composition Tables [27, 28]. Dietary protein intake was also categorized into quartiles to avoid the possible influence of extreme values. Based on previous studies [23, 33], models were adjusted for total energy intake, socio-demographic characteristics (including geographic area, residence, childbearing age, education, occupation, household wealth index, and parity), health-related characteristics (including passive smoking, alcohol drinking, antenatal care visit frequency, folate/iron supplements use, anemia, and medication use), and principal component score based on the nutrient intakes. For birth weight and LBW, models were additionally adjusted for offspring sex and gestational age. Animal protein and plant protein were mutually adjusted for one another. Further adjustment for other major dietary protein sources was performed in the analysis of specific major dietary protein source. To test for a linear trend, we used the median for each quartile of protein intake as a continuous variable. We further evaluated the interactions by introducing cross-product terms into regression models to assess whether the associations were modified by baseline characteristics including offspring sex, geographic area, residence, childbearing age, maternal education, maternal occupation, household wealth index, and parity.

To simulate the substitution of dietary protein for carbohydrate, we fitted isocaloric models [31] by simultaneously including the percentages of energy from fat and protein, total energy intake, and all other potential confounders. The effect estimate from this model can reflect the effect of increasing protein intake at the expense of carbohydrate while keeping calories constant. Similarly, to simulate the substitution of dietary protein for fat, we simultaneously included the percentages of energy from carbohydrate and protein, total energy intake, and all other potential confounders.

A two-tailed *P* < 0.05 was considered as statistically significant. All statistical analyses were performed using STATA software (version 12.0; StataCorp, College Station, Texas, USA).

## Results

### Baseline characteristics

The baseline characteristics of participants by quartiles of total protein and animal protein intakes are present in Table 1. Participants with higher total protein and animal protein intakes tended to be in northern Shaanxi and southern Shaanxi, respectively. Participants with higher total protein and animal protein intakes were more likely to be urban residents, aged 25–29 years at delivery, better educated, work outside, wealthier, at the first delivery, have more than six antenatal care visits, and take folate/iron supplements, and less likely to be exposed to passive smoking. Total protein intake was positively associated with total energy and most nutrient intakes, and negatively associated with fat, monounsaturated fat, polyunsaturated fat, and vitamin E intakes. Animal protein intake was positively associated with total energy and most nutrient intakes, and negatively associated with plant protein, monounsaturated fat, polyunsaturated fat, carbohydrate, and vitamin E intakes. The offspring birth weight and gestational age were 3263 g (SD 440) and 39.6 weeks (SD 1.3), respectively. The results of univariable comparisons suggested significant differences in birth weight and the prevalence of LBW, SGA, and IUGR among quartiles of total protein and animal protein intakes.

**Table 1:** Baseline characteristics of participants by quartiles of total protein and animal protein intakes (*% of energy*) among pregnant women in Shaanxi Province, Northwest China

	Total protein intake (*% of energy*)					Animal protein intake (*% of energy*)				
	Q1	Q2	Q3	Q4	*P*^1^	Q1	Q2	Q3	Q4	*P*^1^
Number of participants	1827	1828	1828	1827		1827	1828	1828	1827	
*General characteristics*										
Geographic area (%)					< 0.001					< 0.001
Northern Shaanxi	36.0	54.8	57.5	61.0		48.6	56.9	53.8	49.9	
Southern Shaanxi	43.6	25.3	22.7	24.3		25.7	22.6	28.1	39.5	
Central Shaanxi	20.4	19.9	19.8	14.7		25.7	20.5	18.1	10.6	
Rural residence (%)	84.2	82.4	74.8	62.9	< 0.001	88.0	83.7	73.6	58.8	< 0.001
Childbearing age (%)					< 0.001					0.012
< 25 years	47.9	43.0	39.0	36.1		43.2	43.0	41.2	38.5	
25–29 years	32.7	37.1	39.1	42.2		35.5	35.9	38.7	41.0	
≥ 30 years	19.5	20.0	21.9	21.7		21.3	21.1	20.2	20.5	
Maternal education (%)					< 0.001					< 0.001
Primary school or below	13.3	8.2	8.1	7.0		15.6	9.0	6.2	5.8	
Junior high school	59.9	59.7	51.4	43.3		60.7	57.9	52.9	42.6	
Senior high school or above	26.9	32.2	41.5	49.8		23.8	33.1	40.9	51.5	
Farmer (%)	78.3	74.3	70.4	62.9	< 0.001	80.9	74.4	69.6	61.0	< 0.001
Household wealth index (%)					< 0.001					< 0.001
Poor	30.4	33.8	30.7	27.1		32.3	34.1	31.6	24.0	
Medium	39.2	38.5	37.0	33.5		40.4	39.0	35.7	33.2	
Rich	30.3	27.7	32.3	39.4		27.3	26.9	32.7	42.9	
Nulliparity (%)	58.1	58.2	58.5	64.6	< 0.001	50.4	58.0	62.1	68.9	< 0.001
Passive smoking (%)	23.8	23.3	19.9	17.8	< 0.001	26.2	21.7	19.3	17.6	< 0.001
Alcohol drinking (%)	1.6	1.2	1.1	1.3	0.522	1.3	0.8	1.4	1.7	0.125
> 6 antenatal check visits (%)	50.4	50.1	56.1	63.5	< 0.001	43.1	49.4	58.3	69.3	< 0.001
Folate/iron supplements use (%)	88.1	89.9	92.0	92.9	< 0.001	86.4	90.3	92.5	94.6	< 0.001
Anemia (%)	19.6	18.3	18.3	17.7	0.494	18.3	20.4	17.6	17.5	0.092
Medication use (%)	19.4	19.8	18.3	18.9	0.711	29.7	19.2	19.0	18.4	0.763
*Nutrient intakes*^b^										
Total energy (kcal/day)	1772.5	2169.2	2424.4	2687.5	< 0.001	1902.1	2217.3	2345.1	2589.2	< 0.001
Protein (% of energy)	8.3	10.7	12.0	14.5	< 0.001	9.5	10.9	11.6	13.7	< 0.001
Animal protein (% of energy)	1.5	2.3	3.0	5.1	< 0.001	0.8	2.0	3.2	6.0	< 0.001
Plant protein (% of energy)	6.8	8.4	9.0	9.4	< 0.001	8.7	8.9	8.4	7.6	< 0.001
Fat (% of energy)	40.0	32.3	32.0	33.3	< 0.001	32.9	32.1	34.7	38.0	< 0.001
Saturated fat (% of energy)	8.2	8.3	9.1	10.3	< 0.001	6.7	8.1	9.6	11.6	< 0.001
Monounsaturated fat (% of energy)	19.4	13.7	12.8	12.4	< 0.001	15.5	13.7	14.3	14.9	< 0.001
Polyunsaturated fat (% of energy)	9.2	7.4	7.1	7.1	< 0.001	8.2	7.6	7.5	7.4	< 0.001
Carbohydrate (% of energy)	49.7	57.4	57.1	53.9	< 0.001	57.2	57.7	54.3	48.9	< 0.001
Vitamin A (μg retinol equivalent/day)	362	333	391	723	< 0.001	349	332	383	745	< 0.001
Thiamin (mg/day)	0.6	0.7	0.7	0.8	< 0.001	0.6	0.7	0.7	0.7	< 0.001
Riboflavin (mg/day)	0.6	0.6	0.7	0.9	< 0.001	0.5	0.6	0.7	0.9	< 0.001
Folate (μg/day)	239	252	291	399	< 0.001	264	277	295	343	< 0.001
Vitamin C (mg/day)	53	57	60	72	< 0.001	57	59	60	62	0.017
Vitamin E (mg/day)	46	40	39	39	< 0.001	42	42	42	41	< 0.001
Calcium (mg/day)	483	528	581	676	< 0.001	472	534	592	669	< 0.001
Iron (mg/day)	28	29	30	31	< 0.001	28	29	29	30	< 0.001
Zinc (mg/day)	5.5	5.6	6.2	7.9	< 0.001	5.4	5.7	6.3	7.7	< 0.001
Selenium (mg/day)	26	27	29	42	< 0.001	25	26	29	44	< 0.001
*Pregnancy outcomes*										
Birth weight (g)	3227.9	3252.0	3266.6	3308.2	< 0.001	3231.7	3262.0	3263.2	3297.9	< 0.001
Gestational age (weeks)	39.6	39.5	39.6	39.6	0.626	39.6	39.6	39.5	39.6	0.361
Sex, male (%)	50.2	53.4	55.6	53.8	0.110	53.1	51.7	54.6	53.7	0.223
Low birth weight (%)	4.3	3.8	2.9	2.2	0.002	4.8	3.0	2.9	2.6	0.001
Small for gestational age (%)	10.9	10.7	10.7	8.4	0.040	12.8	9.9	9.3	8.2	< 0.001
Intrauterine growth retardation (%)	5.1	4.5	4.2	3.0	0.012	5.6	4.3	3.7	3.3	0.004

Values were means or %

^a^*P* values for the differences among groups were derived from χ^2^ tests for categorical variables and analysis of variance for continuous variables

^b^Macronutrient intake was present as a percentage of total energy intake by the nutrient-density method, and other nutrients were energy-adjusted by the residual method

### Status of macronutrient intake

The status of macronutrient intake among pregnant women in Shaanxi, China is shown in Table 2. Dietary protein intake per day in the study population during pregnancy was 66.9 g, which was higher than the recommended level in the first trimester in China (55 g), but lower than the recommended level in the second and third trimesters (70 g and 85 g, respectively). The mean percentages of energy from protein, fat, and carbohydrate were 11.4% (SD 2.2), 34.4% (SD 9.8), and 53.5% (SD 10.6), respectively. The percentages of participants having energy from macronutrient in the recommended ranges were 74.4% for protein, 31.2% for fat, and 58.6% for carbohydrate, with only 18.1% participants having energy from all three macronutrients in the recommended ranges. In particular, 25.2% participants had energy from dietary protein lower than the lower limit of the recommended range 10%. Plant protein accounted for the majority of total protein intake (72.5%), of which cereal was the greatest contributor and provided 46.9% of total protein. Animal protein accounted for only 27.4% of total protein, and the main contributors to animal protein were red meat (34.4%), dairy (23.5%), and eggs (19.7%).

**Table 2:** Dietary macronutrient intake among pregnant women in Shaanxi Province, Northwest China

	Dietary intake (g/day)		Dietary intake (% of energy)	
	Mean	SD	Mean	SD
Dietary protein intake	66.9	31.7	11.4	2.2
Animal protein intake	18.3	7.3	3.0	1.2
Protein intake from red meats^a^	6.3	2.5	1.0	0.6
Protein intake from poultry	2.1	1.1	0.3	0.1
Protein intake from dairy	4.3	2.2	0.7	0.3
Protein intake from eggs	3.6	1.9	0.6	0.2
Protein intake from fish and seafoods	2.1	1.1	0.3	0.2
Plant protein intake	48.5	21.0	8.4	1.9
Protein intake from cereals	31.4	13.6	5.5	1.7
Protein intake from legumes	7.2	4.8	1.2	0.5
Protein intake from nuts	3.4	2.5	0.6	0.3
Protein intake from vegetables and fruits	6.1	3.6	1.0	0.6
Dietary fat intake	85.7	36.5	34.4	9.8
Saturated fat intake	23.5	11.1	9.0	3.4
Monounsaturated fat intake	35.3	14.9	14.6	5.8
Polyunsaturated fat intake	18.9	8.0	7.7	2.6
Dietary carbohydrate intake	311.6	121.2	53.5	10.6

*SD* standard deviation

^a^Red meats included pork, beef, and lamb

### Associations of total protein, animal protein, and plant protein intakes during pregnancy with birth weight, LBW, SGA, and IUGR

**Fig. 1:**
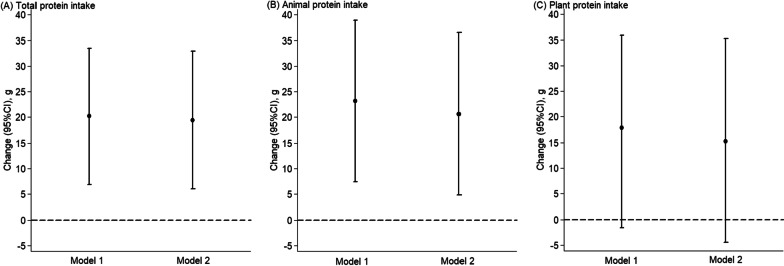
Birth weight changes associated with per 3% increase in energy from dietary protein intake during pregnancy. Multilevel linear regression models were used to estimate changes and 95% CIs. Model 1 was adjusted for total energy intake, offspring sex, gestational age, and socio-demographic characteristics (including geographic area, residence, childbearing age, education, occupation, household wealth index, and parity). Model 2 was adjusted for all variables in Model 1 plus health-related characteristics (including passive smoking, alcohol drinking, antenatal check visit frequency, folate/iron supplements use, anemia, and medication use) and principal component score based on the nutrient intakes. Models were mutually adjusted for animal protein and plant protein. The black circles represent birth weight changes, and the vertical lines represent 95% CIs

The magnitude of change in birth weight associated with dietary protein intake during pregnancy is displayed in Fig. 1. In the full adjusted models, per 3% increase in energy from total protein and animal protein intakes during pregnancy was associated with birth weight increases of 19.4 g (95% CI 6.0–32.9; *P* = 0.004) and 20.6 g (95% CI 4.8–36.5; *P* = 0.009), respectively. However, we observed no significant increase in birth weight associated with plant protein intake during pregnancy.

The mean intakes of total protein during pregnancy in this population were 8.3% and 14.5% of energy in the lowest and the highest quartiles, respectively. After adjusted for all potential confounders, the risks of LBW, SGA, and IUGR were inversely associated with total protein intake (all *P* for trend < 0.05) (Table 3). Compared with the lowest quartile, the adjusted ORs (95% CIs) for the highest quartile of maternal total protein intake were 0.58 (0.36–0.92) for LBW, 0.86 (0.74–0.99) for SGA, and 0.71 (0.54–0.93) for IUGR. Per 3% increase in energy from total protein intake during pregnancy was associated with 22% lower risk of LBW (0.78, 0.64–0.94), 12% lower risk of SGA (0.88, 0.79–0.98), and 16% lower risk of IUGR (0.84, 0.72–0.98). The risks for LBW, SGA, and IUGR were reduced with increasing quartiles of animal protein intake during pregnancy (all *P* for trend < 0.05) (Table 3). Compared with the lowest quartile, the adjusted ORs (95% CIs) for the highest quartile of maternal animal protein intake were 0.48 (0.33–0.72) for LBW, 0.76 (0.61–0.95) for SGA, and 0.71 (0.57–0.88) for IUGR. Per 3% increase in energy from animal protein intake during pregnancy was associated with 21% lower risk of LBW (0.79, 0.65–0.96), 13% lower risk of SGA (0.87, 0.78–0.97), and 14% lower risk of IUGR (0.86, 0.75–0.98). However, we found no significant associations of plant protein intake during pregnancy with LBW, SGA, and IUGR (Table 3).

**Table 3:** Birth outcomes associated with quartiles of dietary protein intake (*% of energy*) during pregnancy

	Mean intake (*% of energy*)	Low birth weight		Small for gestational age		Intrauterine growth retardation	
		Model 1	Model 2	Model 1	Model 2	Model 1	Model 2
Total protein intake							
Q1	8.3	1	1	1	1	1	1
Q2	10.7	0.85 (0.59, 1.23)	0.86 (0.59, 1.24)	0.98 (0.81, 1.18)	0.99 (0.84, 1.17)	0.93 (0.84, 1.03)	0.95 (0.86, 1.05)
Q3	12.0	0.70 (0.47, 1.05)	0.72 (0.48, 1.08)	0.96 (0.79, 1.17)	0.96 (0.80, 1.15)	0.90 (0.80, 1.01)	0.90 (0.80, 1.01)
Q4	14.5	0.56 (0.35, 0.89)	0.58 (0.36, 0.92)	0.86 (0.75, 0.98)	0.86 (0.74, 0.99)	0.69 (0.51, 0.93)	0.71 (0.54, 0.93)
Per 3% of energy		0.77 (0.64, 0.93)	0.78 (0.64, 0.94)	0.87 (0.78, 0.97)	0.88 (0.79, 0.98)	0.84 (0.71, 0.98)	0.84 (0.72, 0.98)
*P*_trend_^a^		0.009	0.011	0.039	0.046	0.03	0.04
Animal protein intake							
Q1	0.8	1	1	1	1	1	1
Q2	2.0	0.58 (0.38, 0.89)	0.62 (0.41, 0.96)	0.79 (0.63, 0.98)	0.81 (0.66, 0.99)	0.79 (0.65, 0.96)	0.79 (0.65, 0.96)
Q3	3.2	0.56 (0.39, 0.82)	0.59 (0.41, 0.87)	0.76 (0.62, 0.93)	0.79 (0.63, 0.98)	0.75 (0.61, 0.92)	0.76 (0.62, 0.93)
Q4	6.0	0.48 (0.33, 0.71)	0.48 (0.33, 0.72)	0.74 (0.58, 0.94)	0.76 (0.61, 0.95)	0.70 (0.55, 0.89)	0.71 (0.57, 0.88)
Per 3% of energy		0.78 (0.65, 0.94)	0.79 (0.65, 0.96)	0.86 (0.77, 0.96)	0.87 (0.78, 0.97)	0.85 (0.74, 0.97)	0.86 (0.75, 0.98)
*P*_trend_^a^		0.017	0.024	0.024	0.029	0.041	0.043
Plant protein intake							
Q1	5.9	1	1	1	1	1	1
Q2	8.0	1.03 (0.70, 1.53)	1.01 (0.68, 1.51)	1.06 (0.84, 1.34)	1.06 (0.84, 1.34)	1.02 (0.73, 1.42)	1.04 (0.74, 1.45)
Q3	9.1	0.93 (0.63, 1.36)	0.94 (0.64, 1.39)	1.01 (0.81, 1.27)	1.01 (0.81, 1.27)	1.01 (0.72, 1.43)	1.00 (0.71, 1.42)
Q4	10.7	0.76 (0.49, 1.18)	0.75 (0.48, 1.17)	1.00 (0.78, 1.27)	1.00 (0.79, 1.28)	0.87 (0.60, 1.26)	0.86 (0.60, 1.25)
Per 3% of energy		0.87 (0.69, 1.10)	0.88 (0.69, 1.11)	0.95 (0.83, 1.08)	0.94 (0.82, 1.08)	0.91 (0.75, 1.11)	0.89 (0.73, 1.08)
*P*_trend_^a^		0.295	0.324	0.950	0.941	0.444	0.397

Multilevel logistic regression models were used to estimate ORs and 95% CIs. Model 1 was adjusted for total energy intake and socio-demographic characteristics (including geographic area, residence, childbearing age, education, occupation, household wealth index, and parity). Model 2 was adjusted for all variables in Model 1 plus health-related characteristics (including passive smoking, alcohol drinking, antenatal check visit frequency, folate/iron supplements use, anemia, and medication use) and principal component score based on the nutrient intakes. Models were mutually adjusted for animal protein and plant protein. Models for low birth weight were additionally adjusted for offspring sex and gestational age

^a^*P* for trend was calculated using the median intake of each quartile as a continuous variable

### Associations of major dietary protein sources during pregnancy with birth weight, LBW, SGA, and IUGR

**Fig. 2:**
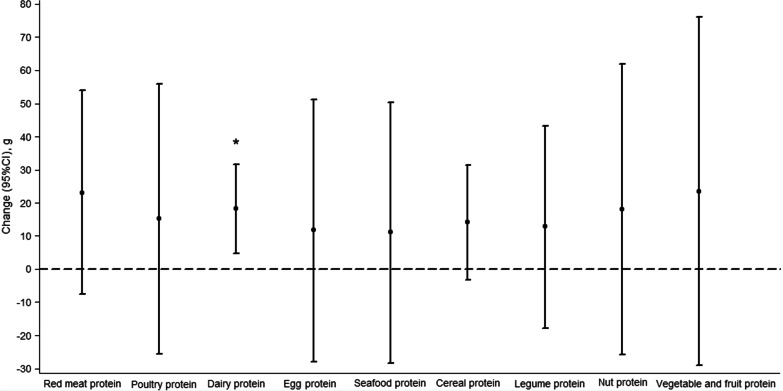
Birth weight changes associated with per 3% increase in energy from major dietary protein sources during pregnancy. Multilevel linear regression models were used to estimate changes and 95% CIs. Models were adjusted for total energy intake, offspring sex, gestational age, socio-demographic characteristics (including geographic area, residence, childbearing age, education, occupation, household wealth index, and parity), health-related characteristics (including passive smoking, alcohol drinking, antenatal check visit frequency, folate/iron supplements use, anemia, and medication use), principal component score based on the nutrient intakes, and mutually adjusted for other major dietary protein sources. The black circles represent birth weight changes, and the vertical lines represent 95% CIs. *P = 0.008

**Fig. 3:**
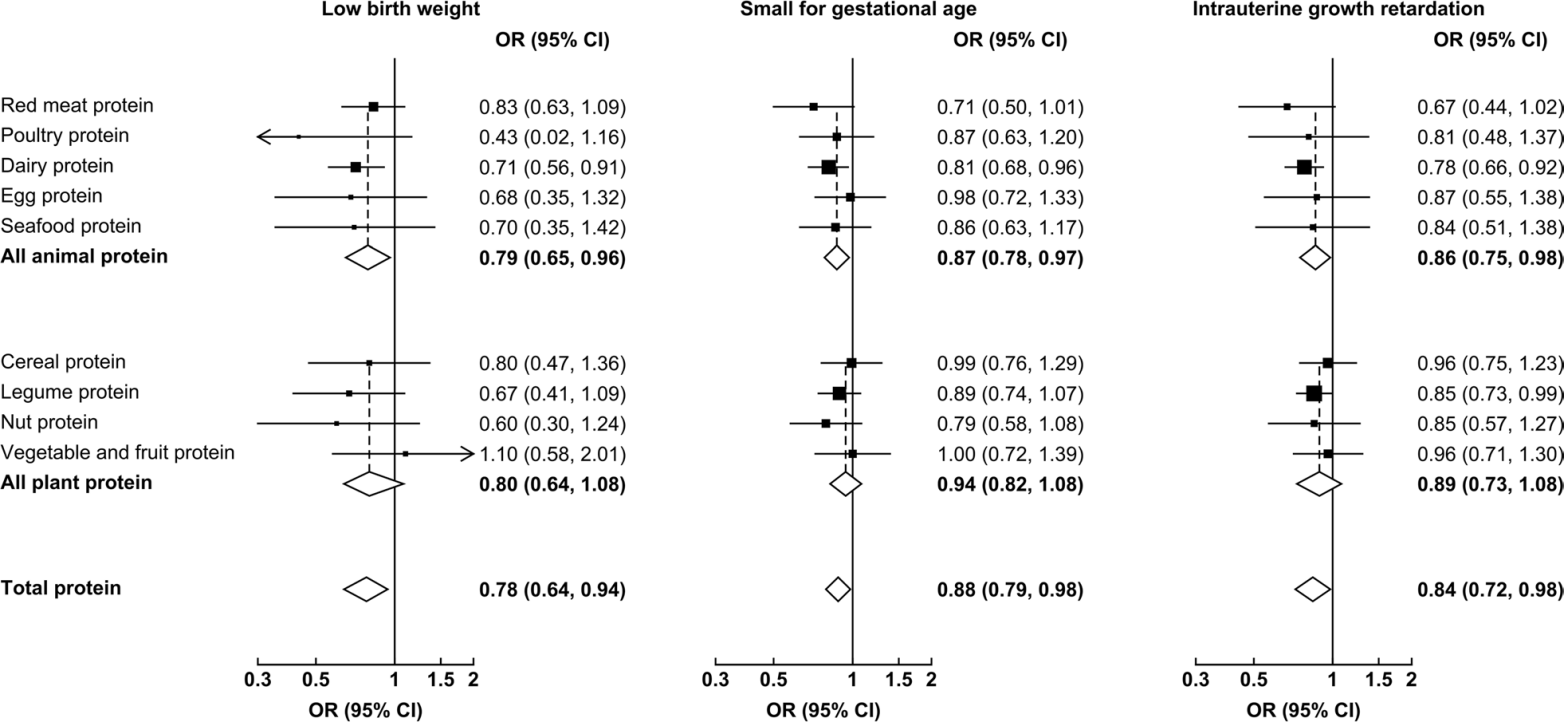
Birth outcomes associated with per 3% increase in energy from major dietary protein sources during pregnancy. Multilevel logistic regression models were used to estimate ORs and 95% CIs. Models were adjusted for total energy intake, socio-demographic characteristics (including geographic area, residence, childbearing age, education, occupation, household wealth index, and parity), health-related characteristics (including passive smoking, alcohol drinking, antenatal check visit frequency, folate/iron supplements use, anemia, and medication use), principal component score based on the nutrient intakes, and mutually adjusted for other major dietary protein sources. Models for low birth weight were additionally adjusted for offspring sex and gestational age. The black boxes represent ORs, with the size inversely proportional to the variance of the logarithm of the OR, and the horizontal lines represent 95% CIs

The magnitude of change in birth weight associated with major dietary protein sources during pregnancy is present in Fig. 2. After adjusted for all potential confounders, per 3% increase in energy from dairy protein intake during pregnancy was associated with an increase of 18.2 g (95% CI 4.7–31.7; *P* = 0.008) in birth weight. The associations of major dietary protein sources during pregnancy with LBW, SGA, and IUGR are shown in Fig. 3. After adjusted for all potential confounders, per 3% increase in energy from dairy protein intake during pregnancy was associated with 29% lower risk of LBW (0.71, 0.56–0.91), 19% lower risk of SGA (0.81, 0.68–0.96), and 22% lower risk of IUGR (0.78, 0.66–0.92). However, there were no significant associations of other major dietary protein sources during pregnancy with birth weight, LBW, SGA, and IUGR.

### Roles of modifiable factors

The associations of different dietary protein sources (total protein, animal protein, plant protein, and major dietary protein sources) during pregnancy with birth weight, LBW, SGA, and IUGR were not significantly modified by baseline characteristics including offspring sex, geographic area, residence, childbearing age, maternal education, maternal occupation, household wealth index, and parity (all *P* for interaction > 0.05).

### Substitution analyses

Substituting 3% energy from carbohydrate with total protein, animal protein, and dairy protein was associated with birth weight increases of 18.9 g (95% CI 4.7–33.2), 20.6 g (95% CI 4.8–36.5), and 19.3 g (95% CI 5.9–32.7), respectively (Additional file 1: Table S1). Substituting 3% energy from carbohydrate with total protein, animal protein, and dairy protein was also associated with lower risks of LBW (total protein: 0.74, 0.59–0.92; animal protein: 0.72, 0.56–0.93; dairy protein: 0.79, 0.63–0.99), SGA (total protein: 0.87, 0.77–0.98; animal protein: 0.86, 0.75–0.98; dairy protein: 0.85, 0.73–0.99), and IUGR (total protein: 0.81, 0.68–0.98; animal protein: 0.84, 0.72–0.98; dairy protein: 0.76, 0.64–0.90) (Additional file 1: Table S1). However, no significant results were observed for the substitution of energy from carbohydrate with plant protein. Similar results were found when energy from protein was exchanged for fat in the associations with birth weight, LBW, SGA, and IUGR (Additional file 1: Table S1).

## Discussion

In our Chinese population with low intake of protein, we observed that higher intake of dietary protein, in particular animal protein, was associated with higher birth weight and lower risks of LBW, SGA, and IUGR. Among specific food sources of protein, higher protein intake from dairy during pregnancy was associated with higher birth weight and lower risks of LBW, SGA, and IUGR. We did not find any significant associations of plant protein and other major dietary protein sources during pregnancy with birth weight, LBW, SGA, and IUGR.

### Comparison with other studies

Previous human studies on dietary protein intake during pregnancy and fetal growth have generally focused on total protein, and the results were not consistent [4–15]. Similar to the present study, several studies in Spain [4], Australia [5], and the UK [6, 7] showed a positive association between maternal protein intake and birth weight, while other studies found no significant association [8–11], an inverse association [12–14], or even an inverse U-curve association [15]. The inconsistency may be partly due to the differences in study designs, dietary assessment methods, and population characteristics such as dietary habits and genetic backgrounds. In particular, the average percentage of dietary protein intake from energy among Chinese pregnant women in the current study was 11.4%, which was much lower than that among pregnant women in Australia (16.3%)[14], the UK (15.7%) [11], and Malay (15.2%) [9]. According to one survey in rural western China, dietary animal protein intake was 12.1 g for males and 8.3 g for women, and occupied less than 20% of total protein [34]. Which was similar to the low animal protein intake in the present study among pregnant women in Shaanxi, Northwest China. Dietary protein intake per day in our study population during pregnancy was higher than that value reported among women in rural western China (40.1 g) [34], indicating that women in pregnancy tended to improve dietary habits of protein intake. One meta-analysis of randomized controlled trails indicated that providing balanced protein energy supplementation (i.e. supplements in which protein provided less than 25% of total energy) resulted in higher birth weight and lower risk of SGA, especially among undernourished populations [35], which were consistent with our findings in the present study.

Previous evidence have suggested that protein actions may vary by the amino acid types and food sources [19, 20]. However, to date, little is known about the relationship between maternal dietary protein intake from different sources, beyond total protein intake, and fetal growth. To our knowledge, only two published human studies have evaluated maternal animal and plant protein intakes separately in association with birth weight [6, 21]. One study involving 538 women in the UK reported a positive association between animal protein intake in late pregnancy and birth weight, but no data was presented about plant protein [6]. More recently, another study involving 1698 women in the Netherlands observed a significantly higher birth weight associated with higher intakes of total protein and animal protein, but not plant protein, among those with the BMI of 17.1–21.2 kg/m^2^ at preconception [21], which were consistent with our findings among Chinese population in the current study. Moreover, only a few studies in Western countries have evaluated the association of maternal protein intake from specific food sources with fetal growth [6, 22, 36]. Similar to the current study, previous studies in Denmark [22], the Netherlands [36], and the UK [6] found a positive association between dairy protein intake during pregnancy and birth weight. Dairy protein among Chinese pregnant women in this study was mainly derived from low- and full-fat milk, yogurt, and formula milk. One study in the UK reported a positive association between meat protein intake in late pregnancy and birth weight [6], while this association was not significant in the present study. The discrepancy may be partly explained by the fact that meat protein intake was much lower among Chinese pregnant women than that in the UK (8.4 vs. 28.3 g/day) [6].

### Possible mechanisms

Adequate protein intake during pregnancy is crucial to support the synthesis of fetal and placental tissues. However, the biological mechanism by which maternal protein influences fetal growth is unclear. Animal studies have shown that offspring born to protein-restricted mothers had lower insulin-like growth factor concentration [37, 38], which was related with suboptimal fetal growth [39]. Pregnancies complicated by IUGR were reported to be characterized by reductions in both cord plasma amino acid concentrations and placental amino acid transporter activity [40].The different effects of animal protein and plant protein on fetal growth may be attributable to the difference in amino acid composition. Animal protein can provide all nine indispensable amino acids, while plant protein can be deficient in one or more indispensable proteins such as lysine or threonine. The deficiency in certain indispensable amino acids may repress protein and lipid syntheses through rapamycin (mTOR) pathway [41], which is important for human growth [42]. Dairy protein is a rich source of indispensable amino acids, and may exert more pronounced effects on fetal growth in our population with such low intake of animal protein. The different effects of animal protein and plant protein on fetal growth may also be due to the fact that pregnant women with higher intake of animal protein may have a much better baseline protein status, such that their subsequent increase in animal protein intake may have raised protein levels enough to be beneficial. In fact, participants with a higher intake of animal protein in the current study tended to be have better overall nutritional status as shown in Table 2.

### Strengths and limitations

To our knowledge, the present study is the first investigation of the associations between dietary protein intake from different sources during pregnancy and fetal growth in Asian countries. This study was conducted in Shaanxi Province of Northwest China using a stratified multistage random sampling method, with the large sample size accounting for 3% neonates in Shaanxi, China. The findings of this study could be generalized to other parts of Northwest China according to the similarities in economy, culture, lifestyle, and dietary habits in these regions and could also partly reflect the status in China. Another strength of this study was the relatively accurate birth outcomes obtained by reviewing birth certificates and medical records. However, some limitations merit discussion. First, the dietary and non-dietary information in pregnancy was retrospectively self-reported by the mothers after delivery. Although previous studies suggested that nutrient intakes and events in pregnancy could be recalled rather well even after years [43–45], we cannot rule out the possible misclassification due to recall bias. For example, the FFQ has a tendency of overestimating the food intake. To minimize bias, we have made efforts to help mothers provide accurate responses during the survey. For one thing, we used standard questionnaires and detailed supporting materials such as food portion images and calendars to collect information. For another thing, we conducted a pilot study to test the survey instruments and trained interviewers rigorously according to the detailed guides before the formal survey. Second, we cannot reveal a real causal association because of the cross-sectional design. Third, we cannot fully exclude the possibility of residual confounding from unobserved and unknown factors even after controlling for many potential confounders, including socio-demographic, health-related and dietary factors. For example, we did not gather information on maternal BMI. According to one meta-analysis involving studies in China, low maternal BMI increased the risks of LBW and SGA, and high maternal BMI was associated with fetal overgrowth [46]. Fourth, we did not collect blood samples, and could not further consider the effects of amino acids status and genetic backgrounds on fetal growth.

## Conclusions

In conclusion, findings from the current study suggest that, among Chinese pregnant women with low intake of protein, higher intake of dietary protein, in particular animal protein and dairy protein, is associated with higher birth weight and lower risks of LBW, SGA, and IUGR. Large-scale prospective cohort studies are needed to evaluate the effects of amino acids or protein from specific sources on fetal growth in broader populations. Future studies with information on body amino acids or protein status and genetic backgrounds are also warranted to further investigate their associations and to identify underlying mechanisms.

## Supplementary Information

**Additional file 1: Fig. S1.** Flow diagram of sampling strategy with exclusion criteria.** Table S1**. Birth weight and the related adverse birth outcomes associated with isocaloric substitution of 3% energy from dietary protein intake during pregnancy.

## Data Availability

The datasets analysed during the current study are available from the corresponding author on reasonable request.

## Abbreviations

LBW: Low birth weight
SGA: Small for gestational age
IUGR: Intrauterine growth retardation
FFQ: Food frequency questionnaire
