# Supplementary material for: Dietary protein intake during pregnancy and birth weight among Chinese pregnant women with low intake of protein
Source: Nutr Metab (Lond). 2022 Jul 5;19:43. doi: 10.1186/s12986-022-00678-0 (PMC9254546; doi:10.1186/s12986-022-00678-0)
Supplement: Supplementary file 1 — Additional file 1: Fig. S1. Flow diagram of sampling strategy with exclusion criteria. Table S1. Birth weight and the related adverse birth outcomes associated with isocaloric substitution of 3% energy from dietary protein intake during pregnancy. [file 12986_2022_678_MOESM1_ESM.docx]

**Figure S1. Flow diagram of sampling strategy with exclusion criteria.**


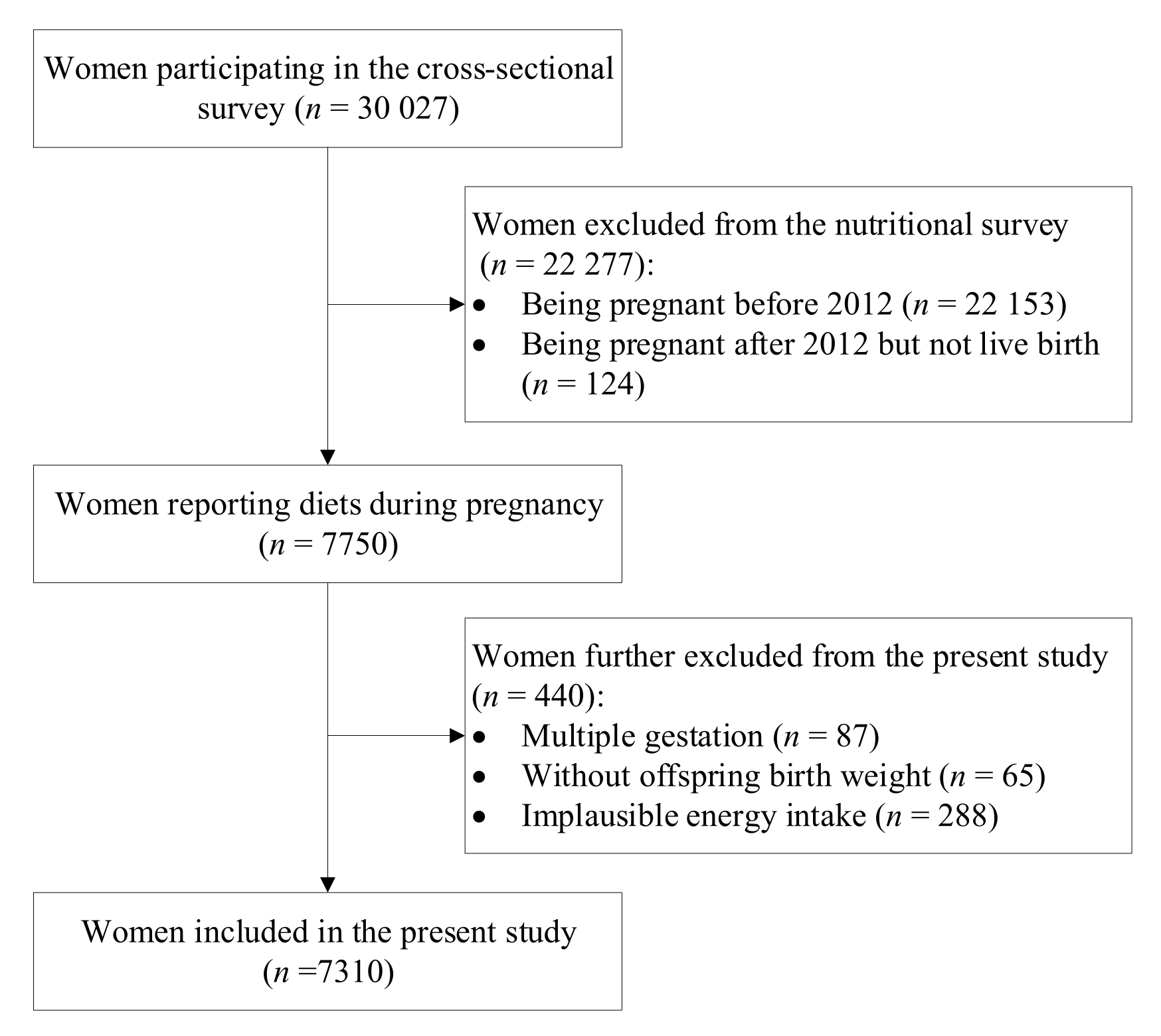


**Table S1. Birth weight and the related adverse birth outcomes associated with isocaloric substitution of 3% energy from dietary protein intake during pregnancy**.

|  | **Birth weight, g** | | **Low birth weight** | | **Small for gestational age** | | **Intrauterine growth retardation** | |
| --- | --- | --- | --- | --- | --- | --- | --- | --- |
|  | Change (95%CI) | *P* | OR (95%CI) | *P* | OR (95%CI) | *P* | OR (95%CI) | *P* |
| **Total protein** |  |  |  | |  |  |  |  |
| Substitution for carbohydrate | 18.9 (4.7, 33.2) | 0.009 | 0.74 (0.59, 0.92) | 0.008 | 0.87 (0.77, 0.98) | 0.025 | 0.81 (0.68, 0.98) | 0.028 |
| Substitution for fat | 19.3 (5.7, 32.9) | 0.005 | 0.75 (0.61, 0.93) | 0.007 | 0.88 (0.78, 0.98) | 0.026 | 0.83 (0.70, 0.98) | 0.035 |
| **Animal protein** |  |  |  |  |  |  |  |  |
| Substitution for carbohydrate | 20.6 (4.8, 36.5) | 0.011 | 0.72 (0.56, 0.93) | 0.013 | 0.86 (0.75, 0.98) | 0.033 | 0.84 (0.72, 0.98) | 0.019 |
| Substitution for fat | 21.1 (4.9, 37.3) | 0.011 | 0.74 (0.57, 0.96) | 0.021 | 0.86 (0.75, 0.99) | 0.043 | 0.84 (0.71, 0.99) | 0.021 |
| **Plant protein** |  |  |  | |  |  |  |  |
| Substitution for carbohydrate | 11.6 (-15.1, 38.4) | 0.393 | 0.71 (0.48, 1.04) | 0.079 | 0.88 (0.71, 1.09) | 0.261 | 0.87 (0.69, 1.07) | 0.242 |
| Substitution for fat | 14.3 (-10.6, 39.2) | 0.262 | 0.69 (0.47, 1.02) | 0.066 | 0.88 (0.72, 1.08) | 0.211 | 0.85 (0.68, 1.06) | 0.132 |
| **Dairy protein** |  |  |  |  |  |  |  |  |
| Substitution for carbohydrate | 19.3 (5.9, 32.7) | 0.013 | 0.79 (0.63, 0.99) | 0.016 | 0.85 (0.73, 0.99) | 0.037 | 0.76 (0.64, 0.90) | 0.021 |
| Substitution for fat | 19.4 (5.7, 33.1) | 0.012 | 0.81 (0.65, 1.00) | 0.019 | 0.86 (0.77, 0.96) | 0.033 | 0.76 (0.65, 0.89) | 0.017 |

Multilevel linear regression models were used to estimated changes and the related 95%CIs, and logistic regression models were used to estimate ORs and the related 95%CIs. Models were adjusted for total energy intake, socio-demographic characteristics (including geographic area, residence, childbearing age, education, occupation, household wealth index, and parity), health-related characteristics (including passive smoking, alcohol drinking, antenatal check visit frequency, folate/iron supplements use, anemia, and medication use), principal component score based on the nutrient intakes, and mutually adjusted for animal protein and plant protein. Further adjustment for other major dietary protein sources was performed in the analysis of dairy protein. Models for birth weight and low birth weight were additionally adjusted for offspring sex and gestational age.
